# Medial Patellofemoral Ligament Reconstruction: Patient-Reported Outcome Measures Comparing Autograft and Allograft Tendons with or Without Tibial Tubercle Osteotomy

**DOI:** 10.3390/jcm14248756

**Published:** 2025-12-10

**Authors:** Eli Beach, Daniel George, Claire Bolton, Shahram Shahrokhi

**Affiliations:** 1 The Queen Elizabeth Hospital (TQEH), Central Adelaide Local Health Network (CAHLN), 28 Woodville Road, Woodville South, SA 5011, Australia; 2 Centre for Orthopaedic and Trauma Research, The University of Adelaide, Adelaide, SA 5000, Australia; daniel.george@sa.gov.au; 3 Sportsmed, 32 Payneham Rd, Stepney, SA 5069, Australia

**Keywords:** medial patellofemoral ligament, patella dislocation, patellofemoral instability

## Abstract

**Objectives:** To compare patient-reported outcome measures (PROMS) as the primary outcome following medial patellofemoral ligament reconstruction (MPFLR) using autograft versus allograft tendon. Secondary objectives were to assess re-dislocation rates and evaluate the effect of concomitant tibial tubercle osteotomy (TTO) on PROMs. **Methods:** Eighty-eight patients from two fellowship-trained orthopaedic surgeons operating at a single institution between 2018 and 2023 were identified. Eligible patients, including those who had undergone an MPFLR with either autograft or allograft +/− TTO, were contacted to complete three validated surveys to quantify PROMS: the Kujala Anterior Knee Pain Score (Kujala), the Marx Activity Score (Marx), and the Norwich Patellar Instability Score (NPI). Exclusion criteria included musculoskeletal or collage disorders and incomplete PROMs. Re-dislocation rates and PROMS were compared between autograft and allograft groups. Independent samples *t*-tests were used, with *p* < 0.05 considered statistically significant. **Results:** A total of fifty-nine patients (46% male, average age 28.5 years old) representing 63 knees returned PROMs. All Kujala scores were similar between groups. Subgroup analysis revealed that patients who received an isolated MPFLR compared to those who received an MPFLR with TTO had lower NPI scores: 22.89% versus 30.21% (*p* < 0.001), respectively. Those who underwent isolated MPLFR with autograft compared to allograft had lower Marx scores: 7.40 versus 7.70 (*p* = 0.031), respectively. One patient who underwent an allograft experienced a recurrence of their patellar instability. **Conclusions:** There was a low recurrent patella dislocation rate following MPFLR and similar anterior knee pain scores in our study when comparing autograft with allograft.

## 1. Introduction

Lateral patella dislocations are common knee injuries especially in the younger, active population and may lead to long-term morbidity [1]. Following a lateral patella dislocation, regardless of the outcome, approximately forty percent of people do not return to their pre-injury sporting level [2]. Complications can include anterior knee pain, subjective instability, recurrent dislocations, and patellofemoral osteoarthritis [3].

The medial patellofemoral ligament (MPFL) is the primary soft tissue restraint of lateral translation of the patella [4]. Mechanisms for patella dislocation include twisting injuries of the knee, such as participating in sport, or as a result of a direct blow to the knee [5]. The patella is most commonly dislocated laterally. Although the cause of lateral patella dislocation varies, the MPFL is damaged in 94% of cases [2]. Following lateral patella dislocation, management can either be non-operative or operative [6].

For isolated first-time lateral patella dislocations, non-operative management is the first-line treatment. This may include physical therapy, muscle strengthening, and bracing [6]. The outcomes following non-operative management for isolated, first-time lateral patella dislocation are generally favourable. If the initial lateral patella dislocation is associated with damage to other structures of the ipsilateral knee, or if the individual develops recurrent patella dislocations, operative management may be indicated [7].

A common approach to surgical repair for lateral patella dislocation includes MPFL reconstruction (MPFLR) [8]. There are different tissue options available for use in ligament reconstruction, namely autograft or allograft. The most common autografts include the use of a free gracilis, or semitendinosus, graft from the patient’s ipsilateral leg, or a quadriceps’ tendon turn down. Options for allograft include various prepared cadaveric tendons which can be irradiated or non-irradiated [9]. If there are concurrent risk factors for recurrent dislocation or failure of MPFLR, a tibial tubercle osteotomy may be performed during surgery in order to distalise and/or medialise the tibial tuberosity [9]. These risk factors may include trochlear dysplasia, patella alta, a tibial tubercle to trochlear groove (TT-TG) distance of more than 20 mm or ligamentous laxity [9].

Understanding which graft option provides better subjective outcomes and reduced recurrent dislocation rates is multifactorial. Currently, there is a consensus in the literature that autograft tendon provides superior results with regard to failure rate for anterior cruciate ligament reconstruction (ACLR) over allograft [10]. Yet, the use of autograft tendon for ligament reconstruction in the ACLR population has been associated with donor-site pain and reduced knee flexion strength following the harvesting of gracilis tendon in addition to semitendinosus for ACLR [11]. The MPFL, however, is not an intraarticular ligament and therefore, the argument of autograft use over allograft may not be directly applicable. If the morbidity of autograft harvest can be avoided without increasing recurrence rate, then this may be advantageous.

Reviews on the topic have yet to provide conclusive results recommending further studies [12,13]. For example, a study by Hendawi, Godshaw, Flowers, Stephens, Haber and Waldron [14] reported higher Kujala Anterior Knee Pain scores indicating less subjective anterior knee pain in the allograft group when compared to the autograft group. This counters the findings of Kumar, Bastrom, Dennis, Pennock and Edmonds [15], who report higher Kujala Anterior Knee Pain scores following autograft reconstruction. For graft failure, current data suggests that allograft tends to have a lower failure rate compared to autograft following MPFLR [12].

Currently, there is no uniformly agreed-upon graft choice for MPFLR. Therefore, this study aimed to compare MPFLR with autograft to MPFLR with allograft, analysing how this related to patient-reported outcomes measures (PROMS). To do this, we utilised three validated surveys as follows: (1) Kujala Anterior Knee Pain Score [16], (2) Marx Activity Score [1,17], and (3) Norwich Patellar Instability Score (NPI) [18]. Furthermore, little research has compared isolated MPFLR being a soft tissue procedure for patella stabilisation to MPFLR with TTO which offers a technique for altering osseous anatomy to improve patella stability. Lastly, we analysed recurrence rates of lateral patella dislocation following patella stabilisation surgery. We hypothesise that there will be similar PROMS and recurrent lateral patella dislocations rates following MPFL reconstruction with either autograft or allograft.

## 2. Methods

### 2.1. Study Design and Patients

**Figure 1:**
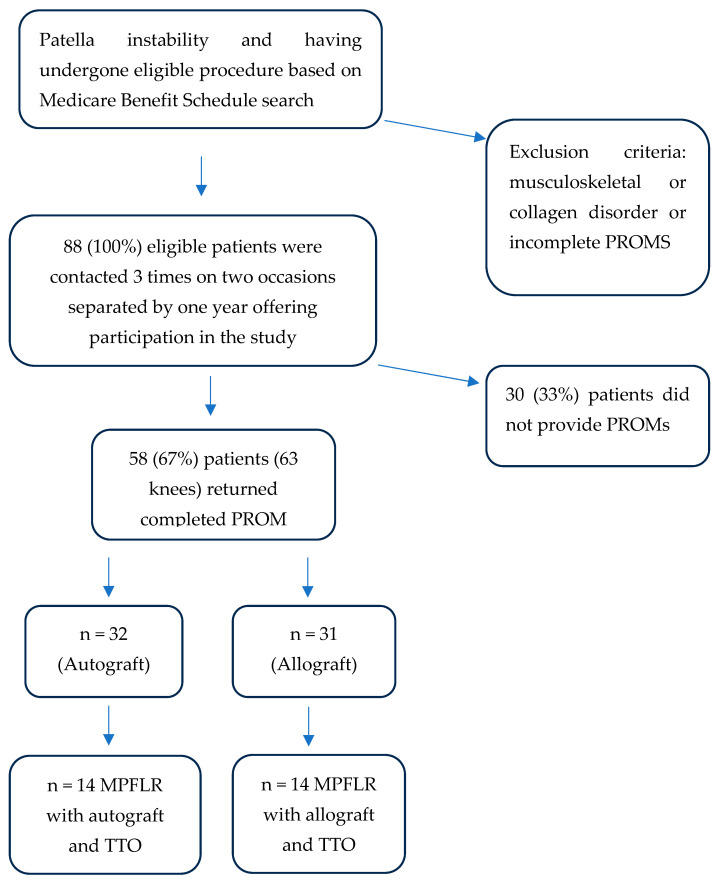
Patient inclusion criteria and subgroup allocation.

This study was a retrospective cohort study. Ethics approval was obtained through the local regulatory body, under approval number 16609. Eighty-eight patients were identified as having patella instability and having undergone eligible procedures based on a Medicare Item Number search. Eligible operations included MPFLR with or without concurrent TTO (Figure 1). Exclusion criteria included patients with a neuromuscular or collagen disorder and failure to complete all three PROMS. Patient age and gender were collected from medical records. Operations were carried out by two fellowship-trained orthopaedic surgeons between May 2018 and June 2023. Demographics including patient age, gender, and operation data including date of surgery, graft choice, operation duration (tourniquet time or operation time) were collected.

#### 2.1.1. Surgical Technique

For this study, two fellowship-trained orthopaedic surgeons (C.B. and S.S.) undertook the same method for MPFL reconstruction, utilising either autograft or allograft for their MPFLR operations and performing a TTO when indicated. The choice to use autograft or allograft tissue was made by the patient following a combined decision-making process considering the risks and benefits of each option. The operative surgeons’ autograft preference in this study was ipsilateral gracilis grafts. Allograft choice was that of non-irradiated cadaveric tibialis anterior or extensor hallucis longus tendons. The tibial tubercle was medialised if the patients had a TT-TG greater than 20 mm, as measured using Computerised Tomography (CT) or Magnetic Resonance Imaging (MRI) (understanding MRI underestimates TT-TG [7]). A medialisation was also undertaken if the TT-TG was between 12 mm and 20 mm, with concurrent risk factors, such as trochlear dysplasia, or ligamentous laxity. In the setting of patella alta, if a Catton Deschamps (CD) ratio of ≥1.3 was achieved, a TT distalisation was performed. If there were excessive TT-TG and patella alta, a combined distalisation and medialisation procedure was performed.

Initially, patients underwent a diagnostic ipsilateral knee arthroscopy to assess for concurrent intracapsular and/or osseous pathology. Repair to injured associated structures was undertaken depending on the findings. If a TTO was indicated, the tibial tubercle was exposed and osteotomised. It was then distalised and/or medialised and secured with k-wires and its position was confirmed with Image Intensifier (II), then secured with two cortical screws with or without a staple, and its final position was confirmed with II. An inlay technique was used for MPFL reconstruction using two-interosseous tunnels in the patella. For the femoral tunnel, fluoroscopy was used to identify Schottles point, and a check for isometricity was undertaken. Femoral fixation was undertaken at 30 degrees of knee flexion with polyether ether ketone (PEEK) screws. Following graft placement, the patella position was assessed arthroscopically during final fixation of the MPFL. This method of MPFLR is consistent with current practice [9].

A standard rehabilitation process was undertaken following surgery, which allowed weight bearing as tolerated. Crutches were offered until quadricep control was achieved, allowing ambulation without an antalgic gait. Follow-up occurred two, six, and twelve weeks post-operatively. After 12 weeks, linear activities were allowed to resume and return to sports was generally allowed from the four- to six-month mark.

#### 2.1.2. Data Collection

Following the collection of demographic data, all 88 eligible patients were contacted. This occurred via phone, text message, and/or email; patients were offered the opportunity to participate in the project a minimum of three times on two separate occasions separated by one year. Consent was obtained and PROMS were collected through an emailed, self-completed online survey containing all questions for the three surveys.

### 2.2. Statistical Analysis

Subjects were divided into the following groups: (1) MPFLR with allograft and TTO versus MPFLR with autograft and TTO, (2) all MPFLR with a TTO compared to all MPFLR without a TTO, (3) MPFLR with either graft choice without a TTO versus with a TTO, (4) MPFLR alone comparing autograft and allograft, and (5) those who had less than or more than 2 years follow-up post-operatively. Differences between groups for PROMS were analysed using independent samples *t*-test. All statistics were calculated with SPSS (IBM SPSS Statistics for iOS version 30.0) and alphas were set at *p* < 0.05 to declare statistical significance.

## 3. Results

In total, 59 eligible patients (68%) representing 63 knees returned completed PROMS. The mean duration of follow-up was 2.9 years (SD = 1.35). Of the 59 patients, 32 were female (54.5%) and 27 were male (45.5%), with an average age of 28.5 years (SD = 9.7) at follow-up. For the reconstruction of the MPFL, 32 patients utilised autograft and 31 patients received an allograft (Table 1). The predominant graft tissue used was ipsilateral gracilis tendon in the autograft group and extensor hallicus longus tendon in the allograft group. Further breakdown of the MPFLR groups included those who received a TTO. There were 14 patients in both the autograft and allograft group who underwent a concurrent TTO along with an MPFLR (Table 1). The overall average operative time was 94.3 min versus 76.9 min for the autograft and allograft groups respectively (Table 1). One individual was excluded from the study due to the presence of trochlea dysplasia and genu valgum alignment. There were no statistically significant differences between the patients in terms of age, sex, graft choice, or overall operative times.

Patient-reported outcome measures included the Kujala Anterior Knee Pain Score, Marx Activity Score, and the Norwich Patella Instability Score. For those individuals who underwent an MPFLR with autograft and a TTO (n = 14) compared to those who underwent an MPFLR with allograft and a TTO (n = 14), there were no significant differences (Table 2). Furthermore, we analysed all those who underwent an MPFLR utilising autograft with or without a TTO (n = 32), compared to those who underwent an MPFLR utilising allograft with or without a TTO (n = 31), for which there were also no statistically significant differences (Table 3).

When comparing PROMS for those who had an MPFLR with either autograft or allograft and a TTO (n = 28) compared to those who had an isolated MPFLR with autograft or allograft (n = 35), there was a statistically significant difference in the Norwich Patella Instability Score, being 30.2% ± 12.4 and 22.9% ± 19.1 (*p* < 0.001), respectively (Table 4). There was no statistically significant difference between the Kujala or Marx scores (Table 4).

When comparing those who underwent an isolated MPFLR with autograft (n = 18) versus allograft (n = 15), there was a statistically significant difference in the Marx score, being 7.4 ± 5.0 and 7.7 ± 6.7 (*p* = 0.031), respectively (Table 5). However, statistically significant differences in Marx scores are unlikely to be clinically meaningful, given the established MCID. There was no statistically different score between with Kujala or Norwich scores (Table 5).

Lastly, when comparing the group who had surgery within the last 2 years with those who underwent surgery more than 2 years ago, prior to analysis, there was no statistically significant difference in either score (Table 6).

There was one episode of recurrent instability within our isolated MPFLR-with-allograft group. This represents a 1.6% overall risk of re-dislocation. One patient had TTO screw irritation and elected to have them removed. There were no other complications.

## 4. Discussion

The purpose of this study was to explore PROMs and the recurrence rates of lateral patellofemoral dislocation following MPFLR by comparing autograft to allograft with or without a TTO. It was hypothesised that there would be similar patient-reported outcome measures in both autograft and allograft groups following surgery. There were statistically higher Marx Activity Scores in those who had isolated MPFLR using allograft compared to MPFLR with autograft; however, this is unlikely to be clinically significant. The Minimal Clinically Important Difference (MCID) for the Marx Activity Rating Scale is reported to be 2.5–3 points. We also found that patients who had MPFLR with a TTO reported higher Norwich Instability Scores compared to those who had an isolated MPFLR; currently, there is no MCID established for the NPI. Despite the increased NPI, there were no differences in the Marx and Kujala scores, demonstrating similar activity levels despite a statistically increased risk of instability symptoms. Furthermore, low re-dislocation rates in our allograft group were found.

There are limited reports of patients’ physical activity levels following MPFLR. Previous work by Kumar, Bastrom, Dennis, Pennock and Edmonds [15] and Flanigan, Shemory, Lundy, Stitgen, Long and Magnussen [3] identified no significant difference in activity levels between autograft and allograft groups. These findings are broadly consistent with prior studies; however, discrepancies in activity levels and Kujala scores may reflect differences in cohort age, skeletal maturity, follow-up duration, and surgical technique, highlighting heterogeneity in MPFLR outcomes across populations. Each group in these studies [3,16] had a population size of 15 and 18, respectively. Interestingly, this cohort group reported more anterior knee pain and greater subjective instability symptoms, although the latter two subjective variables were not significantly different. This emphasises that statistical trends may not always equate to meaningful patient experiences. Given that up to 40% of patients failed to return to pre-morbid physical activity levels following lateral patella dislocation [2], the interpretation of small PROM differences requires caution.

Our finding of increased physical activity levels in those who underwent isolated MPFLR with allograft could be explained by the following factors: autograft harvest is a more significant procedure requiring longer operative times and has been associated with reduced hamstring strength and donor-site pain [9].

Patients who underwent MPFLR with a TTO reported higher Norwich Patella Instability Scores compared to those who had isolated MPFLR. This may be explained by anatomical factors that predispose patients to greater patellofemoral instability, such as elevated TT-TG distance or patella alta [9]. However, there was no significant difference in Kujala or Marx scores in this cohort, indicating that subjective instability does not necessarily correlate with functional activity.

In the cohort of this study, a TTO was undertaken when the individual had an MPFL rupture along with a TT-TG of more than 20 mm, patella alta with a CD ratio of 1.3, or a TT-TG between 12 mm and 20 mm, with additional risk factors such as trochlear dysplasia, patella alta, or ligament laxity [9]. This population may present with higher baseline instability and therefore, higher post-operative NPI scores (Table 4) may be expected, making inferences between surgical effect and baseline instability difficult. Kumar, Bastrom, Dennis, Pennock and Edmonds [15], discussed a comparison of autograft and allograft for MPFLR, with 11 out of 59 adolescents having undergone concurrent osteotomies. However, they did not find significant differences in PROMS for those who underwent secondary procedures, with these being concurrent tibial tubercle transfers, lateral releases, or loose body removals. This comparison provides context and suggests that secondary procedures may not substantially affect PROM outcomes.

Given the heterogeneous population with regard to post operative follow-up time, we chose to explore PROMs, comparing those who had surgery within 2 years from PROMs data collection compared to those who had PROMS data collected more than 2 years post-operatively. For this group, there was no significant difference in any PROM data. Furthermore, graft failures were reported to have occurred on average at 13.8 months post-operatively [14]. Patients beyond 2 years may thus be less likely to experience graft failure, but longer-term follow-up is needed to confirm durability.

The Kujala Anterior Knee Pain scores were not significantly different between any groups in this study. These results were consistent with findings from a recent systematic review by Migliorini, Trivellas, Eschweiler, Knobe, Tingart and Maffulli [12]. However, there was heterogenicity in recent studies, highlighting variability in patient populations and surgical techniques, with Kumar, Bastrom, Dennis, Pennock and Edmonds [15] finding higher Kujala scores in their autograft cohort, whilst Hendawi, Godshaw, Flowers, Stephens, Haber and Waldron [14] found higher Kujala scores in their allograft cohort. Neither study achieved clinically significant differences, emphasising that short-term anterior knee pain is likely comparable between graft types. However, long-term follow-up, including imaging correlation, is warranted to assess the development of post-traumatic patellafemoral osteoarthritis [19].

The increased failure rate of allograft in ACL reconstruction [10] has not been shown to correlate to MPFL reconstruction surgery [13]. The risk of re-dislocation was 1.6% in this study, with one patient experiencing recurrent instability symptoms following MPFLR with allograft. Two recent studies demonstrated higher failure rates utilising autograft for MPFLR [14,15], whilst the studies by Flanigan, Shemory, Lundy, Stitgen, Long and Magnussen [3], Matuszewski, Tramś, Ciszewski, Wilczyński, Tramś, Jakubowski, Matuszewska and John [20] found higher re-dislocation rates in the allograft groups. A possible contributing factor to MPFLR failure in this study could be attributed to the fact that the individual was skeletally immature at the time of surgery. Once the individual reached skeletal maturity, they subsequently returned to normal activity levels without the need for revision surgery. Furthermore, factors that possibly contributed to lower re-dislocation rates could be due to an older cohort compared to the population studied by Kumar, Bastrom, Dennis, Pennock and Edmonds [15] and Hendawi et al. [14], which therefore may not represent a homogeneous comparison. These discrepancies highlight the influence of cohort age, skeletal maturity, and surgical technique. In this study, graft failure in a skeletally immature patient suggests that skeletal maturity may influence MPFLR outcomes.

Whilst our sample size is small (N = 63), limiting our re-dislocation rate outcome findings, it is of similar size to recent comparable studies [14,15]. The average follow-up period in this study cohort was 2.87 years. However, three patients returned PROMS from between six to twelve months post-operatively. This may be important as Hendawi, Godshaw, Flowers, Stephens, Haber and Waldron [14] estimated that graft failure typically occurs after 13.8 months. Long-term follow-up is needed to confirm durability of our outcomes and to assess long-term failure rate.

Given that this study showed comparable outcomes in anterior knee pain and subjective instability between the autograft and allograft group, it appears that patients could expect similar results from receiving an allograft for MPFLR whilst preserving their native hamstring. A further advantage of choosing an allograft may also include shorter operative times and avoiding donor-site morbidity [9]. The average operative time was 94.3 min versus 76.9 min for the autograft and allograft groups, respectively. It should be noted that allograft usage is associated with increased cost and in this setting, autografts remain an appropriate option; however, they should be considered for patients with ligamentous laxity and poor collagen.

Strengths of this study included a relatively larger cohort size (N = 63) compared to similar studies [14,15]. Additionally, the current paper used three different PROMS which was only performed by Flanigan, Shemory, Lundy, Stitgen, Long and Magnussen [3], who reported on Knee Injury and Osteoarthritis Outcome Score (KOOS), Marx Activity Score, and the Norwich Patella Instability Score.

Limitations of this study should also be considered. Firstly, the study was a retrospective cohort study with a 33% non-response rate, introducing potential bias and limiting causal inference. Secondly, patient-reported outcomes measures were not collected pre-operatively, preventing assessment of baseline morbidity and post-operative change. Thirdly, the cohort was small and heterogeneous in various ways, including patients’ older average age (28.7 years) when compared to comparable studies, which focused on adolescents [14,15]. Furthermore, the duration from surgery for three patients was not more than 12 months during follow-up at the time of publication, limiting the interpretability of certain outcomes. However, whilst subgroup analysis in duration of >2-year follow-up revealed no difference, statistical power may be limited due to cohort size and procedure heterogeneity. Lastly, unmeasured cofounders such as surgeons’ specific technique, patient anatomy, activity levels, adherence to rehabilitation, and lack of post-operative imaging limit precision and may influence outcomes. All these points restrict the generalisability of the findings. In order to address these limitations, future prospective and randomised studies with baseline PROMs, standardised follow-up, and imaging correlation are needed to validate these findings and determine long-term functional and structural outcomes.

## 5. Conclusions

In conclusion, this study demonstrates comparable PROMs when autograft compared to allograft is used for MPFLR in the short term. Subgroup analysis revealed that patients undergoing MFLR without TTO reported lower subjective instability, and those with isolated MPFLR allograft were more physically active than their autograft counterparts. There was one episode of recurrence of patella instability in a skeletally immature patient in the allograft group. Based on these results, both graft types provide comparably favourable outcomes, and allograft may be a reasonable option for patients wishing to avoid donor-site morbidity or reduce operative time. Further research is required to further elucidate the morbidity of autograft versus the risk of recurrence with allograft.

## Figures and Tables

**Table 1:** Operation descriptives (tibial tubercle osteotomy (TTO), standard deviation (SD), and minutes (min)).

	Autograft (n = 32)	Allograft (n = 31)	*p* Value
TTO (n = 28)	14	14	
Operation time (min ± SD)	94.3 ± 29.4	76.9 ± 25.9	0.330

**Table 2:** Patient-reported outcome measures: Kujala Anterior Knee Pain Score, Marx Activity Score, and Norwich Patella Instability Score for MPFLR with allograft with TTO versus MPFLR with autograft with TTO. Results are expressed as mean ± SD.

	Autograft (n = 14)	Allograft (n = 14)	*p* Value
Kujala (mean ± SD)	81.1 ± 15.3	87.9 ± 15.8	0.880
Marx (mean ± SD)	5.6 ± 5.6	3.6 ± 4.7	0.630
NPI (% ± SD)	26.9 ± 13.3	33.5 ± 10.9	0.360

**Table 3:** Patient-reported outcome measures: Kujala Anterior Knee Pain Score, Marx Activity Score, and Norwich Patella Instability Score for all patients who received MPFLR, comparing those who had autograft to an allograft. Results are expressed as mean ± SD.

	Autograft (n = 32)	Allograft (n = 31)	*p* Value
Kujala (mean ± SD)	84.7 ± 14.9	88.7 ± 14.0	0.990
Marx (mean ± SD)	6.7 ± 5.3	5.9 ± 6.1	0.109
NPI (% ± SD)	23.1 ± 16.9	29.3 ± 16.1	0.552

**Table 4:** Patient-reported outcome measures: Kujala Anterior Knee Pain Score, Marx Activity Score, and Norwich Patella Instability Score for all patients who received an MPFLR and TTO compared to all those who had an isolated MPFLR. Results are expressed as mean ± SD.

	With a TTO (n = 28)	Without a TTO (n = 35)	*p* Value
Kujala (mean ± SD)	84.5 ± 15.6	88.4 ± 13.5	0.337
Marx (mean ± SD)	4.6 ± 5.1	7.6 ± 5.8	0.249
NPI (% ± SD)	30.2 ± 12.4	22. 9 ± 19.1	<0.001

**Table 5:** Differences in PROMs scores for isolated MPFLR autograft compared to allograft. Results presented as means ± SD.

	Autograft (n = 18)	Allograft (n = 15)	*p* Value
Kujala (mean ± SD)	87.4 ± 14.4	89.4 ± 12.8	0.634
Marx (mean ± SD)	7.4 ± 5.0	7.7 ± 6.7	0.031
NPI (% ± SD)	32.8 ± 43.8	47.6 ± 55.0	0.354

**Table 6:** Differences in PROMS for those who had surgery < 2 years from date of returned PROMS compared to those who underwent surgery ≥ 2 years from date of returned PROMS. Results presented as means ± SD. Mean difference confidence interval 95% (Mean Diff (CI 95%)).

	<2 Years (n = 17)	>2 Years (n = 46)	Mean Diff (CI) 95%	*p* Value
Kujala (mean ± SD)	82.2 ± 15.7	88.2 ± 13.9	−5.9 (−14.1, 2.2)	0.870
Marx (mean ± SD)	6.7 ± 5.5	6.1 ± 5.8	0.6 (−2.6, 3.8)	0.967
NPI (% ± SD)	15.8 ± 14.5	30.0 ± 16.0	−14.1% (−23.0, −5.3)	0.780

## Data Availability

The data remain the property of S.S. and will not be available to third parties.
